# The Orientation of Visual Space from the Perspective of Hummingbirds

**DOI:** 10.3389/fnins.2018.00016

**Published:** 2018-01-30

**Authors:** Luke P. Tyrrell, Benjamin Goller, Bret A. Moore, Douglas L. Altshuler, Esteban Fernández-Juricic

**Affiliations:** ^1^Department of Biological Sciences, Purdue University, West Lafayette, IN, United States; ^2^Department of Biological Sciences, State University of New York at Plattsburgh, Plattsburgh, NY, United States; ^3^William R. Pritchard Veterinary Medical Teaching Hospital, School of Veterinary Medicine, University of California, Davis, Davis, CA, United States; ^4^Department of Zoology, University of British Columbia, Vancouver, BC, Canada

**Keywords:** visual field, fovea, binocular vision, birds, hummingbirds

## Abstract

Vision is a key component of hummingbird behavior. Hummingbirds hover in front of flowers, guide their bills into them for foraging, and maneuver backwards to undock from them. Capturing insects is also an important foraging strategy for most hummingbirds. However, little is known about the visual sensory specializations hummingbirds use to guide these two foraging strategies. We characterized the hummingbird visual field configuration, degree of eye movement, and orientation of the centers of acute vision. Hummingbirds had a relatively narrow binocular field (~30°) that extended above and behind their heads. Their blind area was also relatively narrow (~23°), which increased their visual coverage (about 98% of their celestial hemisphere). Additionally, eye movement amplitude was relatively low (~9°), so their ability to converge or diverge their eyes was limited. We confirmed that hummingbirds have two centers of acute vision: a *fovea centralis*, projecting laterally, and an *area temporalis*, projecting more frontally. This retinal configuration is similar to other predatory species, which may allow hummingbirds to enhance their success at preying on insects. However, there is no evidence that their temporal *area* could visualize the bill tip or that eye movements could compensate for this constraint. Therefore, guidance of precise bill position during the process of docking occurs via indirect cues or directly with low visual acuity despite having a temporal center of acute vision. The large visual coverage may favor the detection of predators and competitors even while docking into a flower. Overall, hummingbird visual configuration does not seem specialized for flower docking.

## Introduction

Many species must solve multiple, often competing, sensory tasks as part of their daily lives (Martin, 2017a). Most hummingbird species, for example, have two foraging tactics that present different perceptual challenges. Hummingbirds (Family Trochilidae) are an incredibly diverse group of New World birds, with over 300 species. A large portion of hummingbird diets are based on the acquisition of arthropods (Wagner, 1946; Remsen et al., 1986; Stiles, 1995). Hummingbirds famously hover to feed on flower nectar as well, and this intricate flight behavior, as well as their maneuverability during flight, has made them focal species for many studies of biomechanics and flight control (e.g., Altshuler and Dudley, 2002; Warwick et al., 2012). Studies of hummingbird flight are complemented by a growing interest in hummingbird vision, building on early work demonstrating hummingbird preference for certain colors (Goldsmith and Goldsmith, 1979) and the ecological and evolutionary implications of those visual preferences (Altshuler, 2003; Shrestha et al., 2013; Muchhala et al., 2014). More recent work on visual control of hummingbird flight strengthened the conclusion that vision is highly important for hummingbird behavior. Hovering hummingbirds use body and head movements to minimize perceived global visual motion—or optic flow—as a strategy to maintain stationary position during hovering (Goller and Altshuler, 2014; Ros and Biewener, 2016, but see Dakin et al., 2016).

To collect nectar, a hummingbird must precisely guide its bill into—or dock with—the flower. Docking includes the visual tasks of finding a flower or feeder, aligning the bill, decelerating, and guiding the bill into that opening. Visual information about the rate of expansion of the target flower can be used to control the deceleration component (Lee et al., 1991), similar to landing in pigeons (Lee et al., 1993) or plunge-diving in gannets (Lee and Reddish, 1981). Once docked into a flower, hummingbirds use global visual motion (Goller and Altshuler, 2014; Ros and Biewener, 2016) and perhaps fine details of the flower petals (e.g., hawkmoths; Farina et al., 1994; Kern and Varjú, 1998) to stabilize hovering. Additionally, hummingbirds must complete the dynamic and visually demanding tasks of detecting, tracking, and visually-guiding the capture of moving arthropods.

A recent study presented retinal ganglion cell density maps and eye sizes for five species of hummingbirds, providing essential information for understanding how they orient their visual systems (Lisney et al., 2015). More specifically, hummingbirds have two centers of acute vision per retina that provide four regions of visual space with high visual resolution (Lisney et al., 2015). One region of high ganglion cell density is located close to the center of the retina and has higher visual resolution (i.e., fovea, an invagination of the retinal tissue with a high density of cells) than the other region of high ganglion cell density (i.e., *area*, high density of cells without an invagination of the retinal tissue) located in the temporal portion of the retina (Lisney et al., 2015).

Lisney et al. (2015) did not characterize the specific projection of the centers of acute vision into the visual field and the extent of binocular and lateral vision that hummingbirds possess. Addressing this gap is important because it can provide a better understanding of the visual landmarks that hummingbirds may pay attention to, and their use of vision to facilitate the precise control of the bill as required for docking with a flower or capturing arthropods. Additionally, visual field configuration data, combined with our current knowledge of the retinal anatomy (Lisney et al., 2015), is a key step to predict gaze direction in hummingbirds. In this study, we (a) characterized the configuration of hummingbird visual fields (i.e., size of the binocular and lateral fields and blind area), (b) established the degree of eye movement, (c) corroborated the presence and position of the centers of acute vision in hummingbird retinae, and (d) projected the centers of acute vision into the visual field. Our study shows, for the first time, how specialized parts of the retina (centers of acute vision, binocular vision) may be used for bill control in hummingbirds.

The binocular field is important for precise bill control and object inspection at close distances (Martin, 2009, 2017b; Tyrrell and Fernández-Juricic, 2017a). One mechanism to align the bill within the environment may be to use the binocular visual field, and portions of the retina specialized for high acuity that project into the binocular field. We predicted that hummingbirds would be able to see their own bill tip (i.e., the image of the bill would fall somewhere in the retina). Additionally, we predicted that the hummingbird temporal *area* would project into the binocular field, and more specifically toward the bill tip. Having a region of high visual acuity aligned with the bill tip would be a sensory specialization for accurate control of bill position when foraging for arthropods and docking with flowers. Under this visual configuration, we predicted that hummingbirds would have a low degree of eye movement because there would be little need to move the eyes as key sensory information for visual guidance would be readily available with eyes in the resting position. Alternatively, if hummingbirds could not see their bill tip or if the bill tip would not fall into the temporal area of the retina, we predicted that hummingbirds could move their eyes forward (i.e., convergent movements) to bring the bill tip into view, leading to a high degree of eye movement.

## Methods

Five Anna's hummingbirds (*Calypte anna*) and two rufous hummingbirds (*Selasphorus rufus*) were captured and housed on the University of British Columbia campus, in individual 0.61 × 0.61 × 0.91-m enclosures, and fed *ad libitum* sugar [15 g/100 mL (wt/vol)] or Nektar-Plus [Nekton, 13 g/100 mL (wt/vol)] solution. All procedures were approved by the relevant animal care committees at the University of British Columbia and Purdue University.

We measured the visual field configuration of all five Anna's hummingbirds and both rufous hummingbirds using the ophthalmoscopic reflex technique (Martin, 1984) and a small-diameter visual field apparatus (Pita et al., 2015). Live, fully alert individuals were restrained at the center of the apparatus by placing their body into a grooved foam block and gently holding the wings against the body with a flat nylon strap that also encompassed the foam block. The hummingbird's bill was positioned parallel to the ground by placing the bill into a grooved wire bracket that was suspended in front of the bird. To record the limits of the retinal visual field, an observer (LPT) viewed one of the bird's eyes through a Keeler Professional ophthalmoscope and moved around the perimeter arm of the visual field apparatus until the retinal reflex disappeared. The corresponding angular position on the perimeter arm was then recorded as the margin of that eye's visual field. We were able to readily induce eye movements from hummingbirds by making light snapping or whistling sounds to the sides and back of their heads. We therefore measured the visual field dimensions when the eyes were at rest, the eyes were converged (i.e., eyes fully adducted), and the eyes were diverged (i.e., eyes fully abducted). By rotating the perimeter arm, we took all the aforementioned measurements at 25 elevations around the bird's head, starting 60° below the bill, moving up in 10° increments and ending directly behind the head. We took special care to record the visual field in the plane that intersects the eyes and the tip of the bill to determine if the bill obstructs the visual field (i.e., if hummingbirds are able to see their bill tip).

To measure the angular projection of the optic axis (i.e., the line passing through the center of the cornea and lens), we mounted a white LED onto the side of the ophthalmoscope and turned off the ophthalmoscope light source. In this ophthalmoscope configuration, three Purkinje images were discernable (corresponding to the anterior and posterior cornea surfaces and to the lens). Looking through the viewfinder, the observer moved the ophthalmoscope until all three Purkinje images were aligned, which corresponds to the position of the optic axis.

To map the location of centers of acute vision to different points in the visual field, we extracted retinae and mapped the distribution of retinal ganglion cells following Ullmann et al. (2012). Three of the Anna's hummingbirds were euthanized with an overdose of ketamine and xylazine. We removed the eyes immediately by cutting away the conjunctiva and severing the optic nerve. We then measured the axial diameter and transverse diameter of each eye using a digital caliper. We hemisected the eyes at the ora serrata, removed the vitreous humor, and fixed and stored the retina in the eyecup with 4% paraformaldehyde in 0.1 M phosphate buffered saline (PBS). One retina from each individual was extracted from the eyecup by cutting away the sclera and gently peeling away the choroid. We bleached the extracted retinae in 6% hydrogen peroxide in PBS for ~15 h to clear the pigmented epithelium. We flattened the retinae by making radial cuts, wholemounted them onto gelatinized slides, and stained the retinal ganglion cells with cresyl violet following Ullmann et al. (2012). To correct for shrinkage that may have occurred during the staining process, we took images of the wholemounted retinae before and after staining.

Retinal ganglion cells (RGCs) were counted on each of the three retinae using the Optical Fractionator method within StereoInvestigator software (MBF Bioscience, Williston, VT), the 100x-oil immersion lens on an Olympus BX51 microscope, and an Olympus S97809 camera. We counted retinal ganglion cells within a 50 × 50 μm counting frame and sampled the retina on a grid of 250 × 250 μm squares with an area sampling fraction of 0.04. Our mean (± SE) Schaeffer's Coefficient of 0.016 ± 0.002 indicates that our sampling strategy was appropriate. At the retinal periphery, non-ganglion cell types were excluded based on their soma size, shape, Nissl accumulation, and staining of the nucleus (following criteria detailed in Baumhardt et al., 2014). Near the fovea, we focused through the multiple retinal ganglion cell layers to insure we counted every ganglion cell within the counting frame. We did not estimate spatial resolving power because RGCs corresponding to the fovea are pushed to the rim of the fovea, and using those density values could result in an overestimate of resolving power (Coimbra et al., 2014). We constructed topographic maps of each retina in R (version 3.3.0) following Garza-Gisholt et al. (2014) to visualize the distribution of RGCs and to identify and locate the potential *area temporalis*.

We measured the projection of each *fovea centralis* and *area temporalis* into visual space for Anna's hummingbirds using the following equation: $degrees\;from\;forward\;=\;180\;-\;\left(\frac{s\;\times \;f}{2}+\frac{b+f}{2}\right)$; where *s* is the location of the *fovea centralis* or *area temporalis* on the retina in Cartesian coordinates on a continuous scale from 0 to 1 (see Moore et al., 2012), *f* is the field of view along the horizontal plane for a single eye in degrees, and *b* is the blind area width in degrees. The data for field of view and blind area were collected as part of the visual field measurements described above. We measured the location of the *fovea centralis* and the *area temporalis* relative to the center of the retina following Moore et al. (2012). Briefly, an ellipse was placed around each topographic map so that it overlay the retinal margins. The center of the retina was taken to be the point of intersection of the vertical and horizontal diameters of the ellipse. The distance of the *fovea centralis* and *area temporalis* from the center of the retina was measured to obtain an x-coordinate and a y-coordinate on a Cartesian coordinate system. For the x-coordinate, a value of 0 corresponds to the center of the retina, a value of −1 corresponds to the temporal retinal margin, and a value of 1 corresponds to the nasal retinal margin. For the y-coordinate, a value of 0 corresponds to the center of the retina, a value of −1 corresponds to the ventral retinal margin, and a value of 1 corresponds to the dorsal retinal margin (**Figure 4B**). For example, if the *area temporalis* were to lie halfway between the center of the retina and the temporal retinal margin, the x-coordinate for its location would be −0.5. This method assumes that regions of equal size across the retina subtend equal angles of visual space, which is likely the case in birds (Holden et al., 1987). As a method for secondary confirmation, we also used the R-package Retistruct (Sterratt et al., 2013) to reconstruct the topographic retina maps and to determine the angular position of the retinal specializations. Because we were able to gather visual field and retina data on the same individual birds, we calculated the projections for each individual then took the mean of those projection values, rather than using the mean location and mean visual field parameters. Means ± SE are presented throughout.

## Results

**Figure 1:**
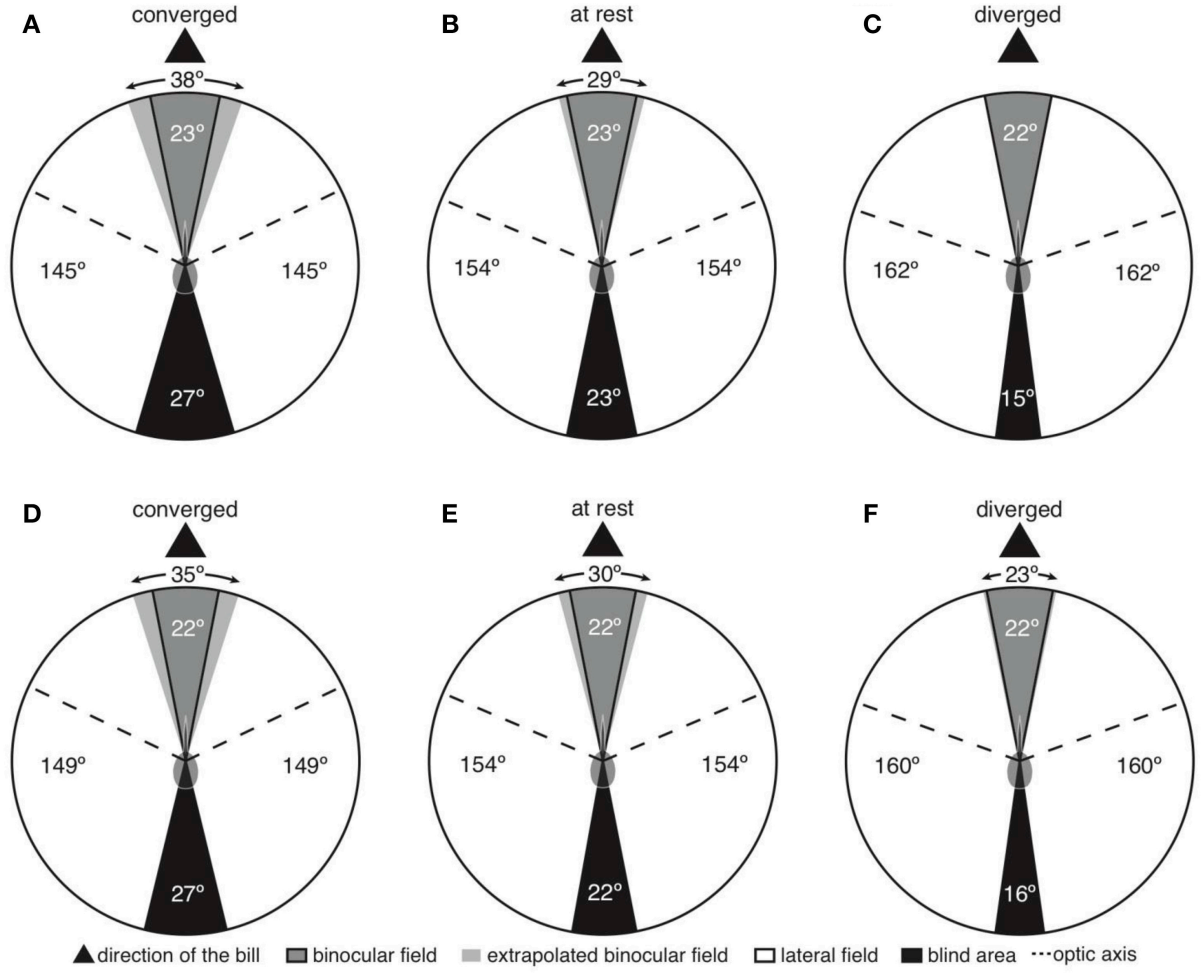
Two-dimensional representations of horizontal sections through the visual fields of Anna's **(A–C)** and Rufous **(D–F)** hummingbirds. Panels are labeled according to whether the panel presents the eyes moved forward in a converged state, resting in an at rest state, or moved backward in a diverged state.

**Figure 2:**
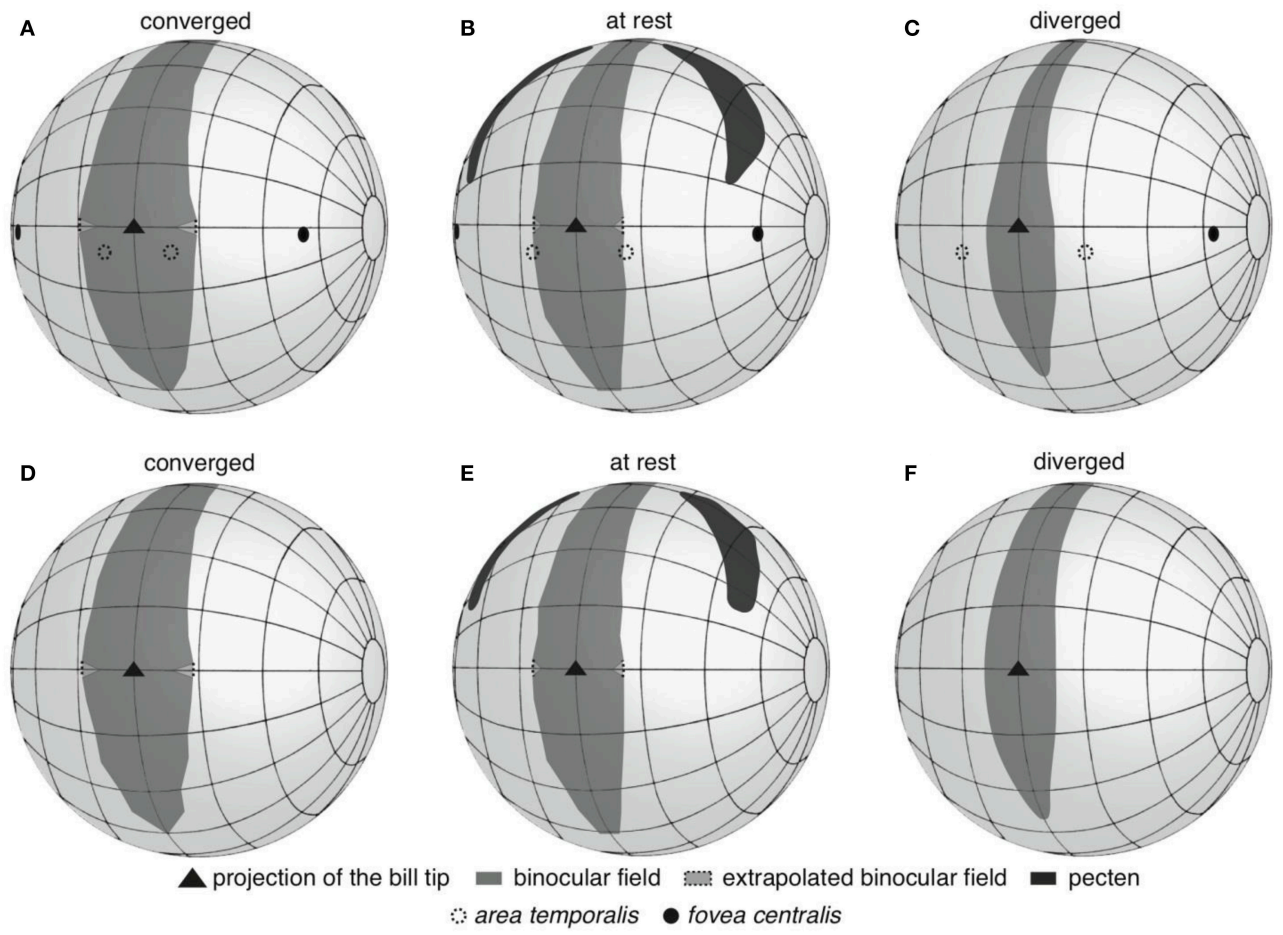
Three-dimensional representations of anterior hemisphere of the visual fields of Anna's **(A–C)** and Rufous **(D–F)** hummingbirds. Panels are labeled according to whether the panel presents the eyes moved forward in a converged state, resting in an at rest state, or moved backward in a diverged state. The projections of retinal specializations are depicted for Anna's hummingbirds; retinal data was not available for Rufous hummingbirds.

Both species shared a very similar visual field configuration (Figure 1), so we present the first set of findings pooling data from Anna's and Rufous hummingbirds. With the eyes at rest in the plane of the bill, the binocular field width was between 22 and 23° (Figures 1B,E); however, the bill was intruding into the binocular field (Figures 1B,E). This means that both species were able to see their own bill tip. In the areas immediately above and below the bill, the binocular field was 29–30° (Figures 2B,E). With the eyes converged in the plane of the bill, the bill was intruding into the binocular field to a larger degree (Figures 1A,D). Thus, above and below the bill, the binocular field increased to 35–38° (Figures 2A,D). Finally, with the eyes diverged, the binocular field in the plane of the bill was not substantially different from the binocular field with the eyes in rest position (Figures 1C,F), because the edge of the binocular field rested right at the bill tip (Figures 2C,F).

The vertical extent of the binocular field with the eyes at rest was 190° (Figures 2B,E). In both species, the width of the binocular field above the head varied between 11–14° (Figures 2B,E), and the binocular field extended 40° behind the top of the head. This pattern was consistent even when the eyes were converged and diverged (Figures 2A,C,D,F). The implication is that hummingbirds can still see above and behind their heads, and the visual fields of the left and right eyes overlap.

The width of the blind area directly behind the head was 22–23°, 27°, 15–16° when the eyes were at rest, converged, and diverged, respectively (Figure 1). Consequently, hummingbirds appear to have a narrow blind area and are able to see ~98% of the celestial hemisphere (eyes at rest).

**Figure 3:**
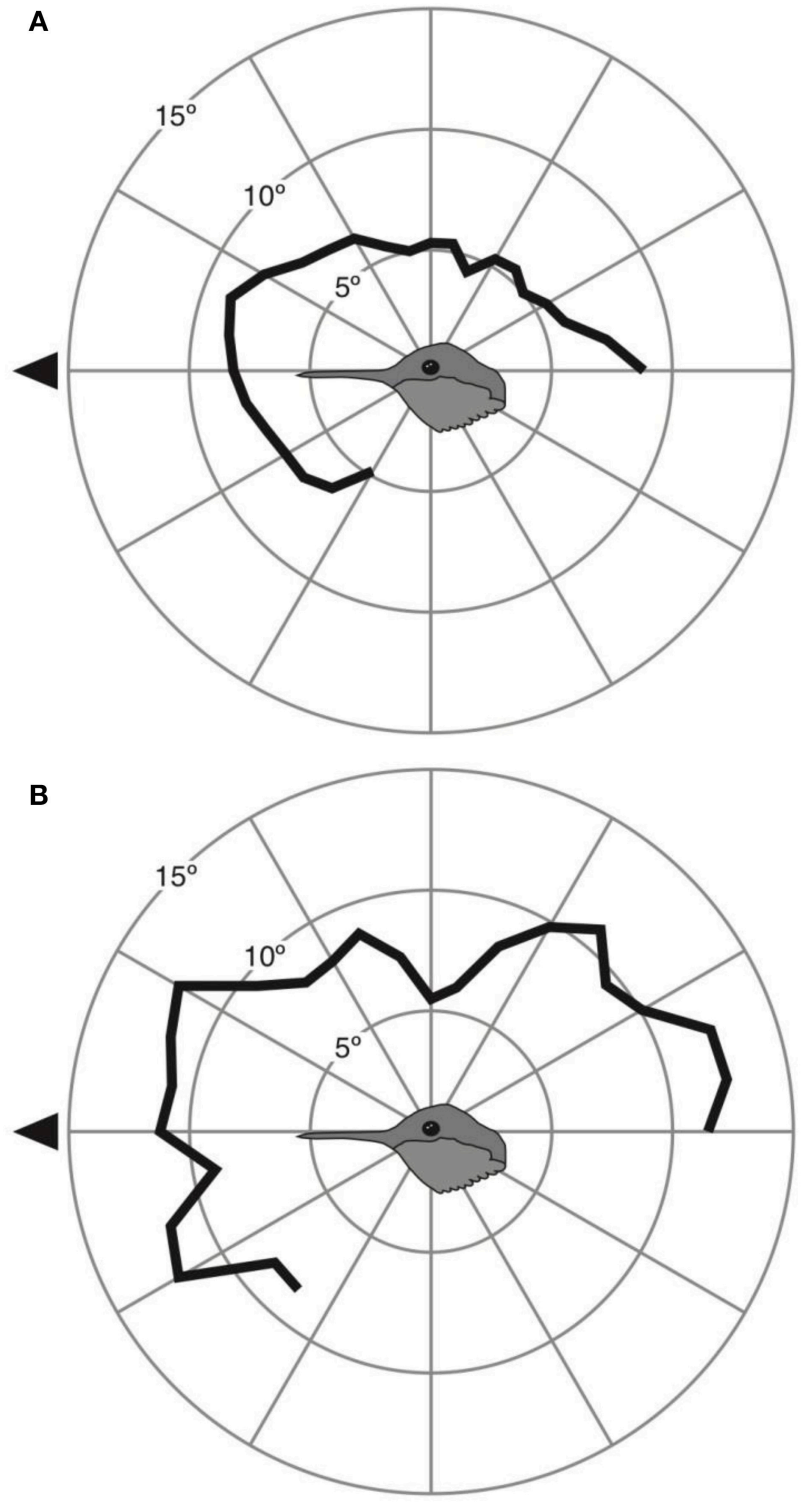
Eye movement magnitude at different elevations around the head for Anna's **(A)** and Rufous **(B)** hummingbirds. Black triangle indicates the direction of the bill.

In terms of eye movements, we found more pronounced between-species differences. Anna's hummingbirds were able to move their eyes a maximum of 9 ± 2° at 20° above the bill (Figure 3A). Rufous hummingbirds were able to move their eyes a maximum of 12 ± 2° at both 30° above and below the bill (Figure 3B).

**Figure 4:**
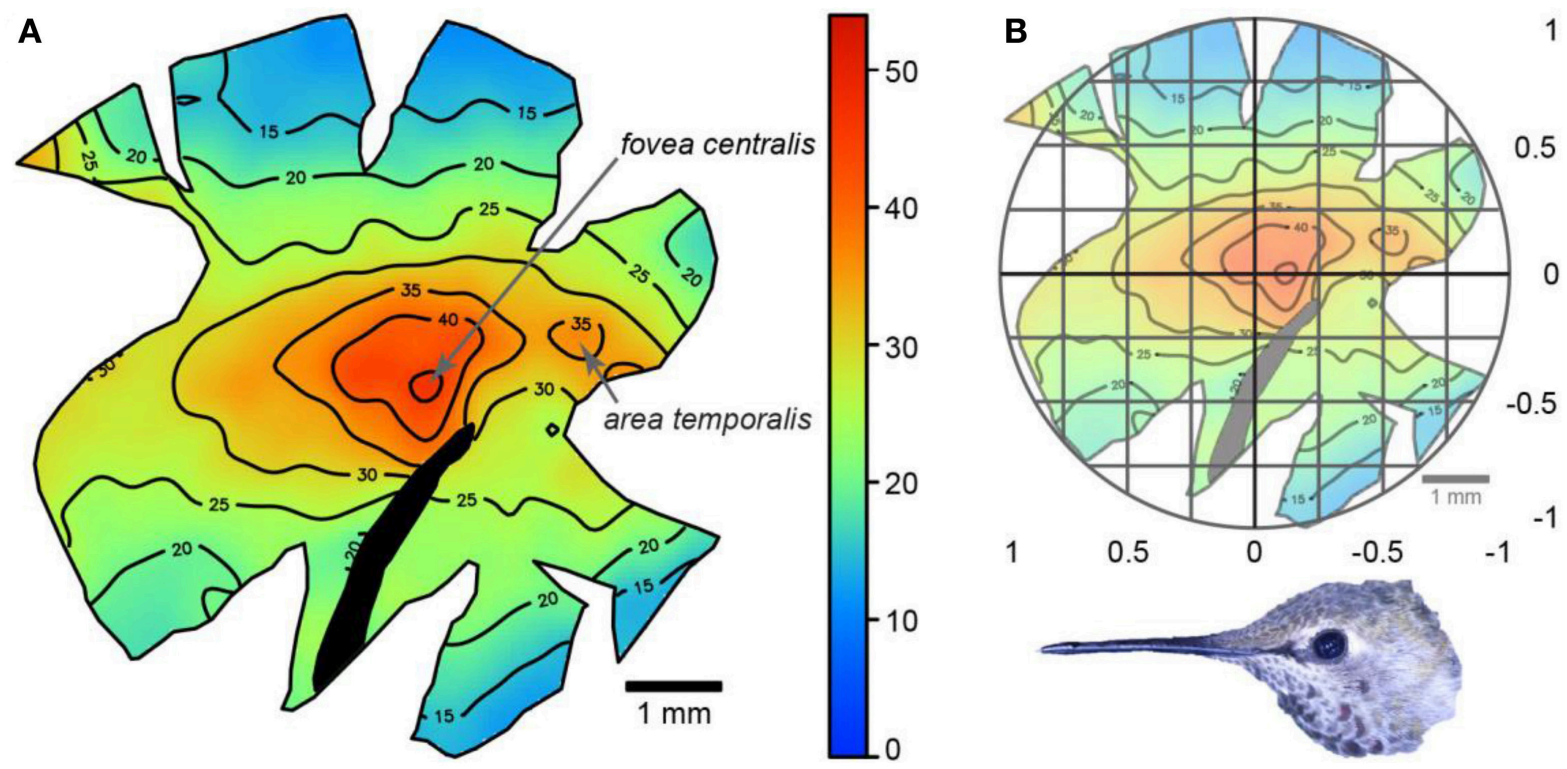
**(A)** Topographic map showing retinal ganglion cell densities across a wholemount of an Anna's hummingbird retina. The numbers represent thousands of cells/mm^2^ and the black section of the retina indicates the position of the pecten. **(B)** The same map with an overlay of the Cartesian coordinate system. Picture shows the relative orientation of the retina relative to the bird's head.

We confirmed the findings of Lisney et al. (2015) by which Anna's hummingbirds had a retina with two centers of acute vision: a *fovea centralis* and an *area temporalis* (Figure 4). The *fovea centralis* was positioned at (−0.20 ± 0.05, 0.04 ± 0.04), and the *area temporalis* at (−0.66 ± 0.07, 0.01 ± 0.01), following the Cartesian coordinate system (Figure 4). Our retinal ganglion cell density estimates were 52,870 ± 2,667 cells/mm^2^ and 40,307 ± 2,004 cells/mm^2^ (peak densities for the fovea and area, respectively). These values are similar to Lisney et al. (2015), who reported Anna's hummingbirds to have retinal ganglion cell densities of 47,619 ± 942 cells/mm^2^ and 38,912 ± 472 cells/mm^2^ for the fovea and area, respectively.

Having information on the visual field configuration and the position of the centers of acute vision allowed us to project the latter into the binocular and lateral fields of Anna's hummingbirds. Using the Cartesian coordinate method, the *fovea centralis* of each eye projected into the lateral field with the eyes at rest (59 ± 4° from forward, 4 ± 3° down), converged (54 ± 4° from forward) and diverged (63 ± 4° from forward; Figures 2A–C). The *area temporalis* of each eye projected into the binocular field when the eyes were converged (8 ± 3° within the margin of the binocular field, but still 12 ± 5° from forward, and 9 ± 0.4° down; Figure 2A), at the margin of the binocular field when the eyes were at rest (2 ± 5° outside the binocular field, or 16 ± 6° from forward; Figure 2B), and into the lateral field when the eyes were diverged (9 ± 7° outside the binocular field, or 20 ± 7° from forward, Figure 2C). It is important to note that despite the *areae temporalis* projecting into the binocular field when the eyes converged, the two *areae* did not intersect on a single viewing point the way that a human's centers of acute vision do. Therefore, Anna's hummingbirds do not appear to use their *areae temporalis* to see their bill tips. The Retistruct method yielded very similar projection estimates as the Cartesian method. Retistruct projected the *fovea centralis* only 3° more laterally and 1° less downward than the Cartesian method. Retistruct also projected the *area temporalis* 4° more forward and 3° less downward than the Cartesian method. The categorical interpretations remained the same between the two methods.

## Discussion

Our findings supported some of our predictions: hummingbirds can see their bill tips with their binocular fields and the temporal *area* projects at the edge of the binocular field or slightly into the binocular field depending on whether the eyes are at rest or converged, respectively. However, other predictions were not supported: the temporal *area* did not project toward the bill tip and the degree of eye movement was not large enough to achieve this. The implication is that visual control of the hummingbird bill tip occurs with the peripheral binocular field despite the presence of a temporal center of acute vision.

Visual control of the bill tip is likely an important factor that determines binocular vision traits in many bird species (Martin, 2009, 2017b; Tyrrell and Fernández-Juricic, 2017a). Hummingbirds have narrower binocular fields relative to some visually guided foragers (e.g., chickadees, crows, sparrows; Troscianko et al., 2012; Moore et al., 2013, 2015), and longer bills relative to their body size. Long bill length could reduce their need for a wide binocular field by increasing the chances of the bill tip falling into the binocular field (Tyrrell and Fernández-Juricic, 2017a). The size of hummingbird binocular fields are consistent with this hypothesis because it is similar in width to other long-billed visually-guided foragers such as starlings, meadowlarks, herons, and cormorants (Martin, 1986; Martin and Katzir, 1994; Martin et al., 2008; Tyrrell et al., 2013).

As Lisney et al. (2015) noted, and we corroborated in the present study, the temporal area is located midway between the central fovea and the temporal margin of hummingbird retinae, rather than at the edge of the retinal margin. As a result of this mid-temporal location, the visual axes of the two temporal *areae* project frontally, yet they do not intersect at a single viewing point (Figure 2). Consequently, hummingbirds do not see their bill tips with the temporal centers of acute vision, even when they move their eyes converging them toward the bill. Although temporal *area* intersection could, in principle, occur with sufficient eye movement, we found that hummingbirds have relatively small degree of eye movements (8–12°) compared to other small avian species (e.g., Tufted Titmouse, ~38°; Song Sparrow, ~36°; Carolina Chickadee, ~35°; American Goldfinch, ~30°; Moore et al., 2013, 2015; Baumhardt et al., 2014). It should also be noted that hummingbirds may still have some other temporal specialization for visually guiding bill-mediated behaviors (e.g., an *area gigantocellularis*; Moore et al., 2017).

Overall, hummingbirds have four centers of acute vision in the frontal hemisphere of their visual field. There are two non-mutually exclusive hypotheses that likely explain this configuration with regards to flower docking. First, it is important for hummingbirds to maintain a consistent position relative to the flower throughout docking, and the high-acuity temporal *areae* would be well positioned to sharply resolve the fine details of flower petals/edges to correct any small changes in relative position. Other sensory information, such as tactile feedback, may also contribute to docked hummingbird position control, but evidence suggests that vision is the dominant sense even after docking (Goller et al., 2017). Another hovering nectar forager, the hawkmoth *Macroglossum stellatarum*, uses a similar visual feedback system. Hawkmoths have highly specialized acute vision directed frontally (Warrant et al., 1999), and stabilize hovering by tracking the movement of pattern edges (Farina et al., 1994; Kern and Varjú, 1998). Second, the retinal configuration of hummingbirds may also be explained by their often-overlooked predatory habits of hawking and gleaning insects (Wagner, 1946). It is a commonly held misconception that hummingbirds are obligate nectarivores. Scientific studies actually show that more than half of hummingbird foraging bouts are predatory bouts (Stiles, 1995); in fact many hummingbirds are digesting arthropods at any given moment in their life cycles (Remsen et al., 1986). Actually, hummingbirds can survive in the wild for long periods of time without access to flowers (Montgomerie and Redsell, 1980). The general retinal configuration of hummingbirds described here is shared with at least two separate bird taxa that take insects in flight: swallows and *Empidonax* flycatchers (Tyrrell and Fernández-Juricic, 2017b).

The central fovea is likely used for long-distance vision while searching for foraging opportunities (Tucker, 2000; Gall and Fernández-Juricic, 2010). During an approach flight, visual motion and image expansion are the most likely important sources of visual information to control behavior (Bhagavatula et al., 2011; Dakin et al., 2016) and acuity may not necessarily be a limiting factor as this type of information does not need to be spatially detailed (Bhagavatula et al., 2011). In the specific case of docking with a flower, visual control may be executed using contra-lateral visual motion and peripheral binocular vision (Martin, 2009) to adjust the position of the bill tip relative to the opening of the corolla. We did not find evidence that hummingbird visual systems have a specialized sensory region that would be directly involved in the visual control of the bill tip with high visual resolution. Consequently, bill position control during docking appears to be similar to other birds that use their binocular field (i.e., low acuity vision) for feeding and/or provisioning the young (Martin, 2017a,b).

Additionally, hummingbirds have relatively narrow blind areas and remarkably comprehensive visual coverage around the head (~98% of the celestial hemisphere). This could become particularly useful as hummingbirds have been documented to suffer from opportunistic predation by other birds, insects, spiders, etc. (e.g., Miller and Gass, 1985; Garcia and Zahawi, 2006; Brooks, 2012; Sazima, 2015) and by high levels of intra- and inter-specific competition (Kodric-Brown and Brown, 1978; Healy and Calder, 2006). By enhancing their visual coverage, hummingbirds would have a higher chance of detecting a threat coming from most directions around the head, thereby allowing for rapid escape as a result of their high flight speeds (e.g., Clark, 2011; Segre et al., 2015; Sholtis et al., 2015).

Once the hummingbird has docked, the large visual field and small blind area may also be important to facilitate detection of competitors and predators. Both hummingbird species described in this study feed from a variety of flowering plants, which have flower corollas of various shapes, sizes, and orientations. For example, rufous hummingbirds are known to feed from buckthorn shrubs (*Rhamnus* spp.), western columbine (*Aquilegia formosa*), scarlet gilia (*Ipomopsis aggregata*), and paintbrushes (*Castilleja* spp.) in sub-alpine meadows (Gass, 1978; Healy and Calder, 2006). Anna's hummingbirds feed from gooseberry (*Ribes speciosum*), chaparral currants (*Ribes malvaceum*), columbines (*A. formosa*), and penstemon (*Keckiella cordifolia*) (Clark and Russell, 2012). Some of these flowers allow the hummingbird to keep the head horizontal while docked, while others require it to be tilted upwards or downwards. With vision covering most of the celestial hemisphere, hummingbirds must be able to monitor the environment above and behind them even though their frontal vision is obstructed due to the bill being inserted into the corolla.

If startled during feeding, hummingbirds have to maneuver to undock. Monitoring the environment for threats and controlling a backward or upward flight maneuver both require hummingbirds to pay attention to motion in parts of the visual field other than the frontal, binocular region. Behavioral evidence suggests that feeding hummingbirds respond to visual motion in various portions of their visual fields (Goller and Altshuler, 2014), and our findings suggest that such visual monitoring reaches most locations behind the head.

In conclusion, hummingbird visual configuration does not seem to show features adapted primarily to the perceptual challenges of flower docking. Hummingbirds may instead use a visual configuration that is common in other bird species (i.e., peripheral binocular vision) for guiding docking into flowers. Ultimately, hummingbirds have retinal features associated with predatory habits (i.e., four centers of acute vision projecting in the frontal part of the head) that may also assist in flight stabilization, but visual field features associated with the detection of approaching predators (i.e., narrow blind areas, large visual coverage).

## Author contribution

All authors listed have made a substantial, direct and intellectual contribution to the work, and approved it for publication.

### Conflict of interest statement

The authors declare that the research was conducted in the absence of any commercial or financial relationships that could be construed as a potential conflict of interest.
